# 4-(4-Meth­oxy­pheneth­yl)-3,5-diphenyl-4*H*-1,2,4-triazole

**DOI:** 10.1107/S1600536812000359

**Published:** 2012-01-14

**Authors:** G. Anuradha, G. Vasuki, Dilek Ünlüer, Emrah Birinci

**Affiliations:** a Department of Physics, Saveetha School of Engineering, Saveetha University, Chennai-5, India; b Department of Physics, Kunthavai Naachiar Government Arts College (w) (Autonomous), Thanjavur-7, India; c Department of Chemistry, Faculty of Arts and Sciences, Karadeniz Teknik University, Trabzon 61080, Turkey

## Abstract

In the title compound, C_23_H_21_N_3_O, the dihedral angles formed by the mean plane of the triazole ring [maximum deviation = 0.007 (1) Å] and the three phenyl rings are 51.13 (8), 52.84 (8) and 47.04 (8)°. In the crystal, mol­ecules are linked by weak C—H⋯N inter­actions, forming infinite chains propagating along the *b*-axis direction.

## Related literature

For details of the synthesis, see: Ünver *et al.* (2011 ▶). For related structures and bond lengths and angles in triazole rings, see: Fun *et al.* (1999 ▶); Gurumoorthy *et al.* (2011 ▶, 2010*a* ▶,*b* ▶); Bruno *et al.* (2003 ▶); Mazur *et al.* (2008 ▶); Sancak *et al.* (2005 ▶).
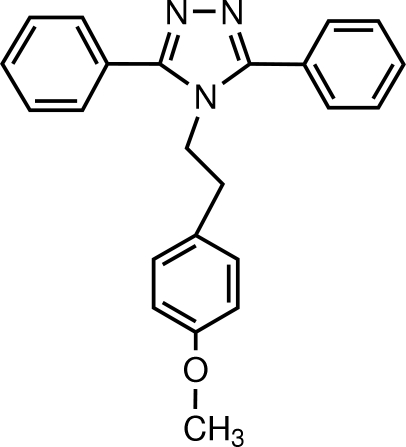


## Experimental

### 

#### Crystal data

C_23_H_21_N_3_O
*M* *_r_* = 355.43
Monoclinic, 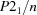

*a* = 13.144 (5) Å
*b* = 7.411 (5) Å
*c* = 21.333 (5) Å
β = 106.835 (5)°
*V* = 1989.0 (16) Å^3^
*Z* = 4
Mo *K*α radiation
μ = 0.07 mm^−1^
*T* = 293 K
0.30 × 0.20 × 0.20 mm

#### Data collection

Bruker Kappa APEXII CCD diffractometer
Absorption correction: multi-scan (*SADABS*; Bruker, 1999 ▶) *T*_min_ = 0.966, *T*_max_ = 0.991
17443 measured reflections
3492 independent reflections
2536 reflections with *I* > 2σ(*I*)
*R*_int_ = 0.037

#### Refinement

*R*[*F* ^2^ > 2σ(*F* ^2^)] = 0.039
*wR*(*F* ^2^) = 0.126
*S* = 1.03
3492 reflections
245 parameters
H-atom parameters constrained
Δρ_max_ = 0.15 e Å^−3^
Δρ_min_ = −0.12 e Å^−3^

### 

Data collection: *APEX2* (Bruker, 2004 ▶); cell refinement: *APEX2* and *SAINT* (Bruker, 2004 ▶); data reduction: *SAINT* and *XPREP* (Bruker, 2004 ▶); program(s) used to solve structure: *SIR92* (Altomare *et al.*, 1994 ▶); program(s) used to refine structure: *SHELXL97* (Sheldrick, 2008 ▶); molecular graphics: *PLATON* (Spek, 2009 ▶) and *Mercury* (Macrae *et al.*, 2008 ▶); software used to prepare material for publication: *SHELXL97*.

## Supplementary Material

Crystal structure: contains datablock(s) I, global. DOI: 10.1107/S1600536812000359/su2362sup1.cif

Structure factors: contains datablock(s) I. DOI: 10.1107/S1600536812000359/su2362Isup2.hkl

Supplementary material file. DOI: 10.1107/S1600536812000359/su2362Isup3.cml

Additional supplementary materials:  crystallographic information; 3D view; checkCIF report

## Figures and Tables

**Table 1:** Hydrogen-bond geometry (Å, °)

*D*—H⋯*A*	*D*—H	H⋯*A*	*D*⋯*A*	*D*—H⋯*A*
C16—H16*A*⋯N2^i^	0.97	2.62	3.542 (3)	160

Symmetry code: (i) 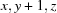.

**Fig. 1.**
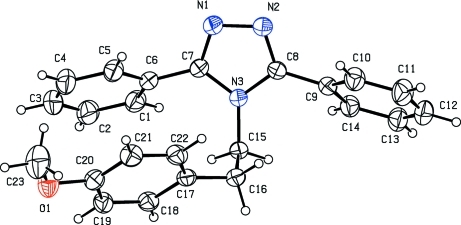
The molecular structure of the title molecule, showing the numbering scheme and displacement ellipsoids drawn at the 50% probability level.

**Fig. 2.**
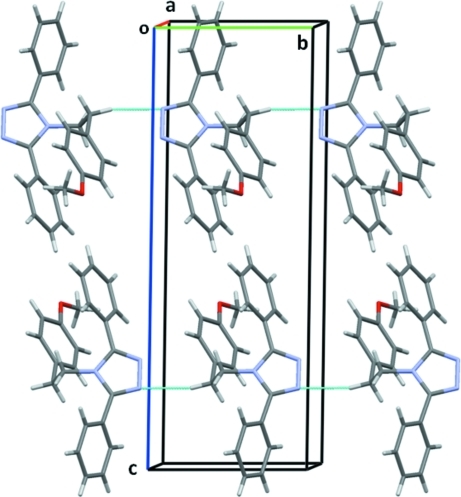
A view along the a axis of the crystal packing of the title compound, showing the C—H···N interactions as dashed cyan lines [see Table 1 for details].

## supplementary crystallographic information

### Comment

The present study is a continuation of our investigations on the structural characterization of 4*H*-1,2,4-triazole derivatives (Gurumoorthy *et al.*, 2011, 2010*a*,*b*). We report herein the crystal structure of the title compound, which was studied to examine the structural activity relationships of a triazole with phenyl substituents.

In the title molecule (Fig. 1) the bond lengths and angles are in agreement with those found for closely related structures, for example, 1-(Benzoylmethyl)-4-(3,5-dimethyl-4*H*-1,2,4-triazol-4-yl)-3- (2-thienylmethyl)-1*H*-1,2,4-triazol-5(4*H*)-one [Sancak *et al.*, 2005], 2-[4-Phenyl-5-(2-pyridyl)-4*H*- 1,2,4-triazol-3-yl]nicotinic acid: a case of solvent-dependent polymorphism [Mazur *et al.*, 2008], and 4-[4-(Dimethylamino)benzylideneamino]-3,5-bis(2-pyridyl)-4*H*-1,2,4-triazole (Bruno *et al.*, 2003)

The title molecule contains four planar rings, namely, a triazole ring A = (N1,N2,C8,N3,C7)] and three benzene rings, B = (C1-C6), C = (C9-C14) and D = (C17-C22). None of the aromatic rings are coplanar with the triazole ring, as observed in the related structure 4-(*p*-Methoxyphenyl)-3,5-bis(2-pyridyl)-4*H*-1,2,4-triazole [Fun *et al.*, 1999]. In the title compound the three phenyl rings (B, C & D) are inclined to the triazole ring (A) by 51.13 (8), 52.84 (8) and 47.04 (8)°, respectively.

The bond angles C6—C7—N3 = 126.29 (14)° and C7—N3—C15 = 127.83 (12)° deviate significantly from the normal value of 120°, and angle N1—C7—C6 = 123.88 (14)° deviates from the normal value of 120°. Torsion angle C9—C8—N3—C15 = 13.1 (2) ° indicates that rings C and D have a *Z*-configuration across the C8—N3 bond. The C7—N3—C15—C16 torsion angle of 85.73 (18)° indicates that the triazole ring and the methoxy phenyl ring is substituted equatorially across the bond N3—C15. Torsion angles N2—N1—C7—C6 = 179.89 (14)° and N2—N1—C8—C9 = 179.40 (14)° indicate that the phenyl rings are substituted *anti*-periplanar to the triazole ring at atoms C7 and C8, respectively.

In the crystal, molecules are linked by a weak C—H···N interaction to form an infinite chain running along the *b* axis direction (Fig. 2).

### Experimental

The compound was synthesized following the published procedure (Ünver *et al.*, 2011)

### Refinement

All the H atoms were positioned in calculated positions and treated as riding on their parent atoms: C—H = 0.93, 0.96 and 0.97 Å for CH, CH_3_ and CH_2_ H atoms, respectively, with U_iso_(H) = k × U_eq_(parent C-atom), where k = 1.5 for CH_3_ H-atoms and k = 1.2 for all other H-atoms.

### Crystal data

**Table:**

C_23_H_21_N_3_O	*F*(000) = 752
*M**_r_* = 355.43	*D*_x_ = 1.187 Mg m^−^^3^
Monoclinic, *P*2_1_/*n*	Mo *K*α radiation, λ = 0.71073 Å
Hall symbol: -P 2yn	Cell parameters from 3242 reflections
*a* = 13.144 (5) Å	θ = 2.5–25.1°
*b* = 7.411 (5) Å	µ = 0.07 mm^−^^1^
*c* = 21.333 (5) Å	*T* = 293 K
β = 106.835 (5)°	Block, colourless
*V* = 1989.0 (16) Å^3^	0.30 × 0.20 × 0.20 mm
*Z* = 4	

### Data collection

**Table:**

Bruker Kappa APEXII CCD diffractometer	3492 independent reflections
Radiation source: fine-focus sealed tube	2536 reflections with *I* > 2σ(*I*)
graphite	*R*_int_ = 0.037
ω and φ scan	θ_max_ = 25.0°, θ_min_ = 2.9°
Absorption correction: multi-scan (*SADABS*; Bruker, 1999)	*h* = −15→15
*T*_min_ = 0.966, *T*_max_ = 0.991	*k* = −8→8
17443 measured reflections	*l* = −25→25

### Refinement

**Table:**

Refinement on *F*^2^	Secondary atom site location: difference Fourier map
Least-squares matrix: full	Hydrogen site location: inferred from neighbouring sites
*R*[*F*^2^ > 2σ(*F*^2^)] = 0.039	H-atom parameters constrained
*wR*(*F*^2^) = 0.126	*w* = 1/[σ^2^(*F*_o_^2^) + (0.0695*P*)^2^ + 0.175*P*] where *P* = (*F*_o_^2^ + 2*F*_c_^2^)/3
*S* = 1.03	(Δ/σ)_max_ < 0.001
3492 reflections	Δρ_max_ = 0.15 e Å^−^^3^
245 parameters	Δρ_min_ = −0.12 e Å^−^^3^
0 restraints	Extinction correction: *SHELXL97* (Sheldrick, 2008), Fc^*^=kFc[1+0.001xFc^2^λ^3^/sin(2θ)]^-1/4^
Primary atom site location: structure-invariant direct methods	Extinction coefficient: 0.0094 (17)

### Special details

**Table:**

Geometry. All e.s.d.'s (except the e.s.d. in the dihedral angle between two l.s. planes) are estimated using the full covariance matrix. The cell e.s.d.'s are taken into account individually in the estimation of e.s.d.'s in distances, angles and torsion angles; correlations between e.s.d.'s in cell parameters are only used when they are defined by crystal symmetry. An approximate (isotropic) treatment of cell e.s.d.'s is used for estimating e.s.d.'s involving l.s. planes.
Refinement. Refinement of *F*^2^ against ALL reflections. The weighted *R*-factor *wR* and goodness of fit *S* are based on *F*^2^, conventional *R*-factors *R* are based on *F*, with *F* set to zero for negative *F*^2^. The threshold expression of *F*^2^ > σ(*F*^2^) is used only for calculating *R*-factors(gt) *etc*. and is not relevant to the choice of reflections for refinement. *R*-factors based on *F*^2^ are statistically about twice as large as those based on *F*, and *R*- factors based on ALL data will be even larger.

### Fractional atomic coordinates and isotropic or equivalent isotropic displacement parameters (Å^2^)

**Table:**

	*x*	*y*	*z*	*U*_iso_*/*U*_eq_	
N3	0.12673 (9)	0.32890 (16)	0.22455 (6)	0.0434 (3)	
N1	0.09718 (11)	0.05004 (17)	0.24966 (7)	0.0533 (4)	
N2	0.10253 (11)	0.05178 (17)	0.18565 (7)	0.0529 (4)	
C8	0.11935 (12)	0.2195 (2)	0.17167 (7)	0.0449 (4)	
C7	0.11099 (12)	0.2166 (2)	0.27187 (7)	0.0456 (4)	
O1	−0.23477 (10)	0.58453 (18)	0.37271 (6)	0.0733 (4)	
C15	0.12614 (13)	0.52730 (19)	0.22388 (8)	0.0479 (4)	
H15A	0.1705	0.5698	0.1977	0.058*	
H15B	0.1564	0.5714	0.2682	0.058*	
C16	0.01528 (13)	0.6037 (2)	0.19644 (8)	0.0520 (4)	
H16A	0.0211	0.7294	0.1853	0.062*	
H16B	−0.0198	0.5402	0.1562	0.062*	
C9	0.13099 (13)	0.2774 (2)	0.10790 (7)	0.0474 (4)	
C17	−0.05381 (13)	0.5917 (2)	0.24178 (8)	0.0472 (4)	
C20	−0.17867 (13)	0.5789 (2)	0.32786 (8)	0.0530 (4)	
C18	−0.02777 (13)	0.6922 (2)	0.29958 (8)	0.0525 (4)	
H18	0.0324	0.7651	0.3096	0.063*	
C6	0.11037 (13)	0.2693 (2)	0.33816 (8)	0.0497 (4)	
C14	0.21927 (14)	0.3726 (2)	0.10374 (8)	0.0571 (5)	
H14	0.2718	0.4041	0.1418	0.068*	
C22	−0.14328 (14)	0.4854 (2)	0.22919 (8)	0.0573 (5)	
H22	−0.1623	0.4166	0.1911	0.069*	
C19	−0.08864 (14)	0.6860 (2)	0.34186 (8)	0.0555 (4)	
H19	−0.0694	0.7540	0.3801	0.067*	
C21	−0.20611 (14)	0.4773 (2)	0.27126 (9)	0.0605 (5)	
H21	−0.2661	0.4042	0.2614	0.073*	
C13	0.22975 (17)	0.4210 (3)	0.04357 (9)	0.0691 (5)	
H13	0.2892	0.4854	0.0411	0.083*	
C10	0.05400 (15)	0.2300 (3)	0.05051 (8)	0.0636 (5)	
H10	−0.0056	0.1654	0.0526	0.076*	
C11	0.06540 (18)	0.2783 (3)	−0.00952 (9)	0.0778 (6)	
H11	0.0138	0.2456	−0.0478	0.093*	
C1	0.19254 (16)	0.3679 (2)	0.37906 (9)	0.0640 (5)	
H1	0.2497	0.4034	0.3645	0.077*	
C5	0.02664 (15)	0.2143 (2)	0.36101 (9)	0.0631 (5)	
H5	−0.0287	0.1467	0.3343	0.076*	
C12	0.15271 (18)	0.3745 (3)	−0.01286 (10)	0.0766 (6)	
H12	0.1598	0.4083	−0.0534	0.092*	
C2	0.1902 (2)	0.4136 (3)	0.44110 (10)	0.0817 (6)	
H2	0.2452	0.4814	0.4681	0.098*	
C4	0.02563 (19)	0.2603 (3)	0.42367 (11)	0.0817 (6)	
H4	−0.0306	0.2236	0.4389	0.098*	
C3	0.1069 (2)	0.3595 (3)	0.46336 (11)	0.0888 (7)	
H3	0.1057	0.3902	0.5054	0.107*	
C23	−0.32829 (18)	0.4804 (4)	0.35991 (12)	0.1021 (8)	
H23A	−0.3601	0.4964	0.3947	0.153*	
H23B	−0.3112	0.3553	0.3570	0.153*	
H23C	−0.3773	0.5184	0.3193	0.153*	

### Atomic displacement parameters (Å^2^)

**Table:**

	*U*^11^	*U*^22^	*U*^33^	*U*^12^	*U*^13^	*U*^23^
N3	0.0484 (8)	0.0397 (7)	0.0433 (7)	−0.0014 (6)	0.0151 (6)	−0.0002 (6)
N1	0.0615 (9)	0.0468 (8)	0.0537 (8)	−0.0044 (6)	0.0198 (7)	0.0018 (6)
N2	0.0626 (9)	0.0452 (8)	0.0525 (8)	−0.0046 (6)	0.0193 (7)	−0.0023 (6)
C8	0.0441 (9)	0.0432 (9)	0.0466 (9)	−0.0005 (7)	0.0120 (7)	−0.0027 (7)
C7	0.0454 (9)	0.0445 (9)	0.0469 (9)	−0.0017 (7)	0.0136 (7)	0.0034 (7)
O1	0.0639 (8)	0.0946 (10)	0.0677 (8)	−0.0174 (7)	0.0292 (7)	−0.0047 (7)
C15	0.0585 (10)	0.0394 (8)	0.0501 (9)	−0.0040 (7)	0.0222 (8)	−0.0015 (7)
C16	0.0648 (11)	0.0426 (9)	0.0480 (10)	0.0041 (8)	0.0157 (8)	0.0041 (7)
C9	0.0542 (10)	0.0436 (9)	0.0447 (9)	0.0013 (7)	0.0148 (8)	−0.0022 (7)
C17	0.0535 (10)	0.0390 (8)	0.0473 (9)	0.0029 (7)	0.0118 (7)	0.0026 (7)
C20	0.0490 (10)	0.0554 (10)	0.0546 (10)	−0.0034 (8)	0.0149 (8)	0.0039 (8)
C18	0.0522 (10)	0.0466 (9)	0.0586 (10)	−0.0092 (7)	0.0159 (8)	−0.0038 (8)
C6	0.0599 (10)	0.0442 (9)	0.0468 (9)	0.0029 (8)	0.0181 (8)	0.0055 (7)
C14	0.0623 (11)	0.0607 (10)	0.0495 (10)	−0.0061 (9)	0.0183 (8)	−0.0045 (8)
C22	0.0621 (11)	0.0514 (10)	0.0539 (10)	−0.0074 (8)	0.0097 (9)	−0.0087 (8)
C19	0.0602 (11)	0.0559 (10)	0.0502 (10)	−0.0092 (8)	0.0159 (8)	−0.0068 (8)
C21	0.0538 (11)	0.0607 (11)	0.0636 (12)	−0.0142 (8)	0.0118 (9)	−0.0043 (9)
C13	0.0838 (14)	0.0710 (12)	0.0600 (12)	−0.0132 (10)	0.0328 (11)	−0.0016 (10)
C10	0.0651 (12)	0.0705 (12)	0.0527 (11)	−0.0093 (9)	0.0128 (9)	−0.0059 (9)
C11	0.0869 (15)	0.0935 (15)	0.0440 (11)	−0.0068 (13)	0.0049 (10)	−0.0029 (10)
C1	0.0781 (13)	0.0606 (11)	0.0509 (11)	−0.0097 (10)	0.0147 (9)	0.0028 (9)
C5	0.0696 (12)	0.0659 (11)	0.0586 (11)	0.0026 (9)	0.0262 (10)	0.0088 (9)
C12	0.1036 (17)	0.0800 (14)	0.0499 (12)	−0.0021 (13)	0.0283 (11)	0.0052 (10)
C2	0.1120 (18)	0.0747 (14)	0.0526 (12)	−0.0107 (12)	0.0145 (12)	−0.0038 (10)
C4	0.0977 (17)	0.0911 (15)	0.0706 (14)	0.0114 (13)	0.0469 (13)	0.0118 (13)
C3	0.136 (2)	0.0829 (15)	0.0526 (12)	0.0136 (15)	0.0356 (14)	0.0005 (11)
C23	0.0752 (15)	0.143 (2)	0.1000 (18)	−0.0381 (15)	0.0436 (13)	−0.0071 (16)

### Geometric parameters (Å, °)

**Table:**

N3—C8	1.3696 (19)	C14—C13	1.378 (2)
N3—C7	1.3697 (19)	C14—H14	0.9300
N3—C15	1.470 (2)	C22—C21	1.386 (2)
N1—C7	1.316 (2)	C22—H22	0.9300
N1—N2	1.3877 (19)	C19—H19	0.9300
N2—C8	1.312 (2)	C21—H21	0.9300
C8—C9	1.476 (2)	C13—C12	1.374 (3)
C7—C6	1.469 (2)	C13—H13	0.9300
O1—C20	1.3672 (19)	C10—C11	1.379 (3)
O1—C23	1.410 (2)	C10—H10	0.9300
C15—C16	1.514 (2)	C11—C12	1.370 (3)
C15—H15A	0.9700	C11—H11	0.9300
C15—H15B	0.9700	C1—C2	1.375 (3)
C16—C17	1.509 (2)	C1—H1	0.9300
C16—H16A	0.9700	C5—C4	1.383 (3)
C16—H16B	0.9700	C5—H5	0.9300
C9—C14	1.382 (2)	C12—H12	0.9300
C9—C10	1.389 (2)	C2—C3	1.373 (3)
C17—C22	1.376 (2)	C2—H2	0.9300
C17—C18	1.395 (2)	C4—C3	1.369 (3)
C20—C21	1.379 (2)	C4—H4	0.9300
C20—C19	1.384 (2)	C3—H3	0.9300
C18—C19	1.369 (2)	C23—H23A	0.9600
C18—H18	0.9300	C23—H23B	0.9600
C6—C1	1.384 (2)	C23—H23C	0.9600
C6—C5	1.388 (2)		
			
C8—N3—C7	104.92 (13)	C17—C22—C21	122.31 (16)
C8—N3—C15	125.81 (12)	C17—C22—H22	118.8
C7—N3—C15	127.83 (12)	C21—C22—H22	118.8
C7—N1—N2	107.74 (12)	C18—C19—C20	120.23 (16)
C8—N2—N1	107.00 (12)	C18—C19—H19	119.9
N2—C8—N3	110.49 (13)	C20—C19—H19	119.9
N2—C8—C9	123.67 (14)	C20—C21—C22	119.31 (16)
N3—C8—C9	125.83 (14)	C20—C21—H21	120.3
N1—C7—N3	109.83 (14)	C22—C21—H21	120.3
N1—C7—C6	123.88 (14)	C12—C13—C14	120.27 (19)
N3—C7—C6	126.29 (14)	C12—C13—H13	119.9
C20—O1—C23	117.67 (15)	C14—C13—H13	119.9
N3—C15—C16	112.31 (13)	C11—C10—C9	120.37 (18)
N3—C15—H15A	109.1	C11—C10—H10	119.8
C16—C15—H15A	109.1	C9—C10—H10	119.8
N3—C15—H15B	109.1	C12—C11—C10	120.08 (18)
C16—C15—H15B	109.1	C12—C11—H11	120.0
H15A—C15—H15B	107.9	C10—C11—H11	120.0
C17—C16—C15	114.86 (13)	C2—C1—C6	120.38 (19)
C17—C16—H16A	108.6	C2—C1—H1	119.8
C15—C16—H16A	108.6	C6—C1—H1	119.8
C17—C16—H16B	108.6	C4—C5—C6	119.85 (19)
C15—C16—H16B	108.6	C4—C5—H5	120.1
H16A—C16—H16B	107.5	C6—C5—H5	120.1
C14—C9—C10	118.91 (15)	C11—C12—C13	120.04 (18)
C14—C9—C8	121.36 (14)	C11—C12—H12	120.0
C10—C9—C8	119.68 (15)	C13—C12—H12	120.0
C22—C17—C18	117.06 (15)	C3—C2—C1	120.2 (2)
C22—C17—C16	123.21 (15)	C3—C2—H2	119.9
C18—C17—C16	119.73 (15)	C1—C2—H2	119.9
O1—C20—C21	124.90 (16)	C3—C4—C5	120.4 (2)
O1—C20—C19	115.60 (15)	C3—C4—H4	119.8
C21—C20—C19	119.50 (16)	C5—C4—H4	119.8
C19—C18—C17	121.58 (15)	C4—C3—C2	120.0 (2)
C19—C18—H18	119.2	C4—C3—H3	120.0
C17—C18—H18	119.2	C2—C3—H3	120.0
C1—C6—C5	119.14 (16)	O1—C23—H23A	109.5
C1—C6—C7	121.75 (15)	O1—C23—H23B	109.5
C5—C6—C7	119.08 (16)	H23A—C23—H23B	109.5
C13—C14—C9	120.32 (17)	O1—C23—H23C	109.5
C13—C14—H14	119.8	H23A—C23—H23C	109.5
C9—C14—H14	119.8	H23B—C23—H23C	109.5
			
C7—N1—N2—C8	−0.02 (17)	N3—C7—C6—C1	−51.7 (2)
N1—N2—C8—N3	0.79 (17)	N1—C7—C6—C5	−50.6 (2)
N1—N2—C8—C9	179.40 (14)	N3—C7—C6—C5	130.15 (17)
C7—N3—C8—N2	−1.21 (17)	C10—C9—C14—C13	0.7 (3)
C15—N3—C8—N2	−168.36 (14)	C8—C9—C14—C13	178.24 (16)
C7—N3—C8—C9	−179.79 (14)	C18—C17—C22—C21	0.3 (2)
C15—N3—C8—C9	13.1 (2)	C16—C17—C22—C21	−179.53 (16)
N2—N1—C7—N3	−0.75 (17)	C17—C18—C19—C20	−0.2 (3)
N2—N1—C7—C6	179.89 (14)	O1—C20—C19—C18	−178.99 (15)
C8—N3—C7—N1	1.19 (17)	C21—C20—C19—C18	0.5 (3)
C15—N3—C7—N1	167.99 (14)	O1—C20—C21—C22	179.03 (16)
C8—N3—C7—C6	−179.46 (14)	C19—C20—C21—C22	−0.4 (3)
C15—N3—C7—C6	−12.7 (2)	C17—C22—C21—C20	0.0 (3)
C8—N3—C15—C16	78.47 (18)	C9—C14—C13—C12	−0.3 (3)
C7—N3—C15—C16	−85.73 (18)	C14—C9—C10—C11	−0.4 (3)
N3—C15—C16—C17	74.66 (17)	C8—C9—C10—C11	−177.97 (17)
N2—C8—C9—C14	−125.15 (18)	C9—C10—C11—C12	−0.4 (3)
N3—C8—C9—C14	53.2 (2)	C5—C6—C1—C2	−1.2 (3)
N2—C8—C9—C10	52.4 (2)	C7—C6—C1—C2	−179.33 (17)
N3—C8—C9—C10	−129.25 (18)	C1—C6—C5—C4	0.8 (3)
C15—C16—C17—C22	−113.57 (18)	C7—C6—C5—C4	178.96 (17)
C15—C16—C17—C18	66.60 (19)	C10—C11—C12—C13	0.8 (3)
C23—O1—C20—C21	−0.7 (3)	C14—C13—C12—C11	−0.5 (3)
C23—O1—C20—C19	178.77 (18)	C6—C1—C2—C3	1.0 (3)
C22—C17—C18—C19	−0.2 (2)	C6—C5—C4—C3	−0.1 (3)
C16—C17—C18—C19	179.62 (15)	C5—C4—C3—C2	−0.2 (3)
N1—C7—C6—C1	127.53 (18)	C1—C2—C3—C4	−0.3 (3)

### Hydrogen-bond geometry (Å, °)

**Table:**

*D*—H···*A*	*D*—H	H···*A*	*D*···*A*	*D*—H···*A*
C16—H16A···N2^i^	0.97	2.62	3.542 (3)	160

Symmetry codes: (i) *x*, *y*+1, *z*.
